# Enhanced recovery after surgery program reduces length of hospital stay and complications in liver resection: A PRISMA-compliant systematic review and meta-analysis of randomized controlled trials

**DOI:** 10.1097/MD.0000000000007628

**Published:** 2017-08-04

**Authors:** Yiyang Zhao, Han Qin, Yang Wu, Bo Xiang

**Affiliations:** a Sichuan University West China Hospital, Pediatric Surgery; b Chengdu First People's Hospital, General Surgery, Chengdu, Sichuan, P.R. China.

**Keywords:** enhanced recovery program, hepatectomy, systematic review and meta-analysis

## Abstract

**Background:**

Many enhanced recovery after surgery (ERAS) guidelines have already been established in several kinds of surgeries. But due to concerns of the specific complications, it has not yet been considered the standard of care in liver surgery.

**Objective:**

The aim of this review is to assess the effect of ERAS in patients undergoing liver surgery.

**Methods:**

EMBASE, CNKI, PubMed, and the Cochrane Database were searched for randomized controlled trials (RCTs) comparing ERAS with conventional care in patients undergoing liver surgery. Subgroup meta-analysis between laparoscopic and open surgical approaches to liver resection was also conducted.

**Results:**

Seven RCTs were included, representing 996 patients. Length of stay (LOS) (MD −3.17, 95% CI: −3.99 to −2.35, *P* < .00001, *I*^2^ = 89%) and time to first flatus (MD −0.9, 95% CI: −1.36 to −0.45, *P* = .0001, *I*^2^ = 98%) were both reduced in the ERAS group. There were also fewer complications in the ERAS group (OR 0.52, 95% CI: 0.37–0.72, *P* < .0001, *I*^2^ = 0%).

**Conclusion:**

The ERAS program can obviously enhance short-term recovery after liver resection. It is safe and worthwhile. A specific ERAS guideline for liver resection is recommended.

## 1 Introduction

Clinical care pathways have developed rapidly over the last decade with the aim of decreasing variability, enhancing quality of care, improving postoperative outcomes, and decreasing health care delivery costs.^[1,2]^ Such multimodal approach is recently referred to as an enhanced recovery after surgery pathway (ERAS) or a fast-track pathway (FT).

Widely adopted for colorectal surgery, ERAS has been shown effective in reducing length of hospital stay (LOS) and complications. While improving patient satisfaction,^[3–5]^ it is still limited applied to liver resection surgery due to concerns of its specific complications, such as postoperative hemorrhage, biliary leakage, and even liver failure. The aim of this meta-analysis was to assess the effect and safety of implementation of ERAS in patients undergoing liver surgery.

## 2 Methods

This analysis is reported on the basis of the PRISMA (Preferred Reporting Items for Systematic Reviews and Meta-Analyses) guidelines.^[6]^ A literature search of EMBASE, CNKI, PubMed, and the Cochrane Database was performed independently by 2 researchers (YZ, HQ) in February 2017. The databases were searched for the period 1997 to 2017. The search terms were grouped in 2 areas: the “ERAS” term (enhanced recovery OR early recovery OR early discharge OR fast track OR fast-track) and the “liver resection” term (liver resection OR liver surgery OR hepatic resection OR hepatic surgery OR hepatectomy). We did not apply language restrictions. All abstracts were reviewed for relevance. The full texts of relevant articles were subsequently reviewed. Ethical approval was not necessary because this study was a systematic review and meta-analysis. Our data were based on published trials only.

### 2.1 Inclusion and exclusion criteria

Only studies that clearly compared ERAS program to non-ERAS program in patients who underwent liver resection were included in the meta-analysis. The study should clearly state the ERAS program, which should contain at least 4 items of ERAS components mentioned above. If more than 1 study was reported by the same institute, only the most recent or higher level study was included. Reviews without original data, and case reports were excluded. Studies that lacked a control group or compared ERAS program in both arms were also excluded.

### 2.2 Outcomes of interests

Outcomes of interests were LOS, time to first flatus, and occurrence of any complication within 30 days postoperatively.

Hospital stay was defined as the interval from the day of surgery to the day of actual discharge from the hospital. Postoperative complications included any complication from the time of surgery to 30 days after discharge according to the Clavien–Dindo classification.^[7]^

### 2.3 Data extraction

The following parameters were extracted from each study by 2 authors (YZ, HQ) independently: year of study publication, study country, study type, patient demographics, ERAS protocols, discharge criteria, and study quality.

### 2.4 Quality assessment

The qualities of RCTs were assessed according to the Cochrane Collaboration tool^[8]^ for assessing risk of bias, which analyzes the following criteria: random sequence generation; allocation concealment; blinding of participants and personnel; blinding of outcome assessment; incomplete outcome data; selective reporting; and other bias. All disagreements were resolved by discussion until a consensus was achieved.

### 2.5 Statistical analysis

This meta-analysis was performed in line with recommendations from the Cochrane Collaboration^[9]^ and the Quality of Reporting of Meta-analyses^[10]^ guidelines. All statistical analyses were performed by using Review Manager Version 5.0 (The Cochrane Collaboration, Oxford, United Kingdom). *I*^2^ values were used for quantification of statistical inconsistency, defined as the percentage of variation between studies due to heterogeneity.^[11]^ And a value exceeding 50% was considered to represent significant heterogeneity. A random-effects model was used to report the results of heterogeneous data; otherwise a fixed-effects model was used. Odds ratios (ORs) with Mantel–Haenszel method were used as a summary measure of efficacy for dichotomous data while mean differences (MDs) with inverse variance method were applied for continuous variables. A 95% confidence interval (CI) was reported for both measures. If the study provided medians and ranges instead of means and standard deviations, the means and standard deviations were imputed, as described by Hozo et al.^[12]^ Forest plots were constructed, and the value of *P* < .05 was considered to indicate statistical significance.

### 2.6 Subgroup analysis

Considering variability of surgical techniques (laparoscopy or open surgery), we included a meta-analysis of subgroups for outcomes affected by this technical difference, including LOS, and postoperative complications.

## 3 Results

### 3.1 Eligible studies

**Figure 1:**
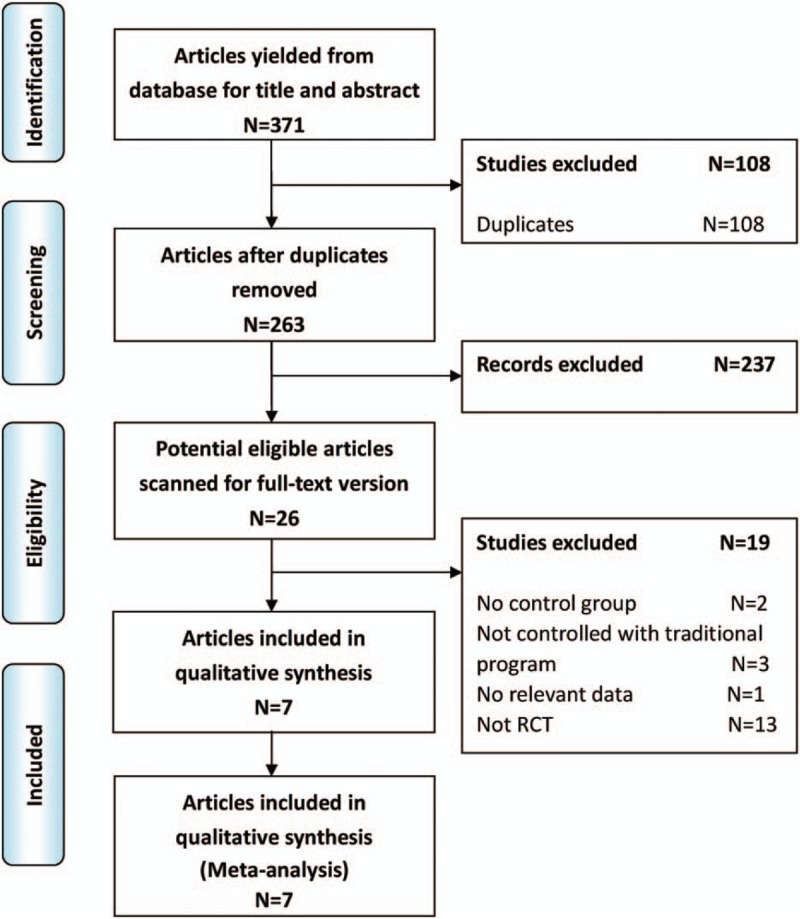
PRISMA flow diagram of literature search.

The literature search yielded 371 studies initially. The PRISMA diagram is shown in Fig. 1. After removing duplicates, the titles and abstracts of 263 articles were reviewed. Of these, 239 were not related to ERAS in liver resection, 2 did not have a control group,^[13,14]^ 3 were not controlled with traditional program,^[15–17]^ 1 did not have relevant data,^[18]^ and 13 were not RCTs.^[2,19–30]^ Finally, this left a total of 7 studies for inclusion in the meta-analysis.^[31–37]^

### 3.2 Study characteristics

The characteristics of included studies are presented in Table 1. The 7 RCTs included 996 patients: 482 in the ERAS group and 514 in the Control group. Three studies were conducted by laparoscopy^[31,33,37]^ and 4 by open surgery.^[32,34–36]^ All studies explicitly described an ERAS protocol. The individual components are summarized in Table 2. Five studies^[31–33,35,37]^ mentioned certain discharge criteria and they were mainly concentrated on good pain control, tolerance of solid food, and independent mobilization.

**Table 1:**
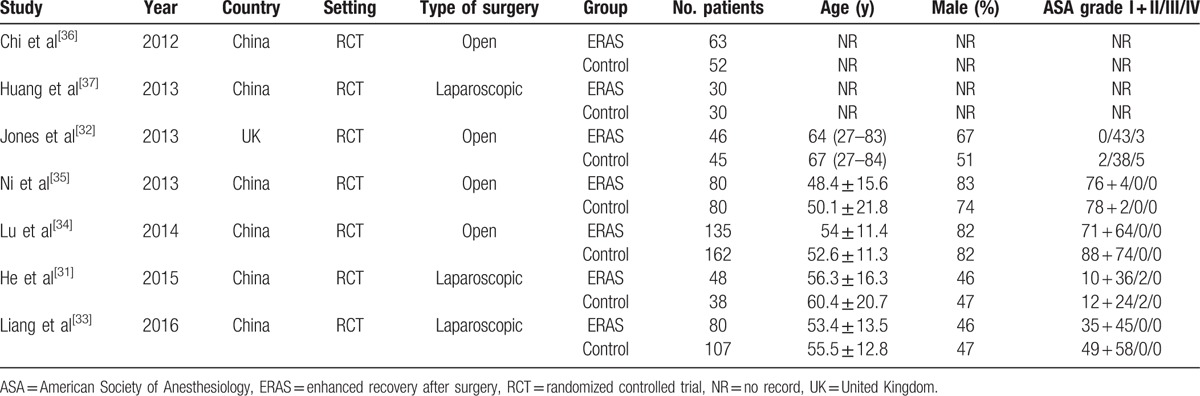
Characteristics of included studies.

**Table 2:**
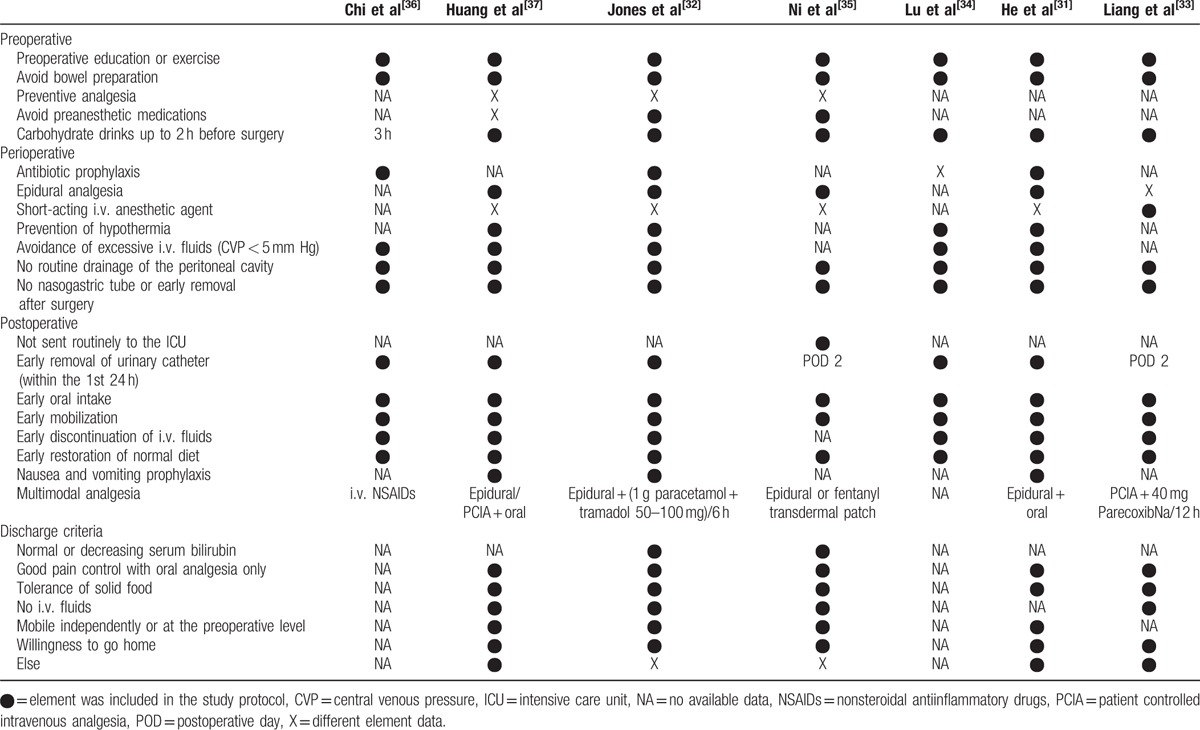
Summary of enhanced recovery program.

### 3.3 Quality assessment

**Figure 2:**
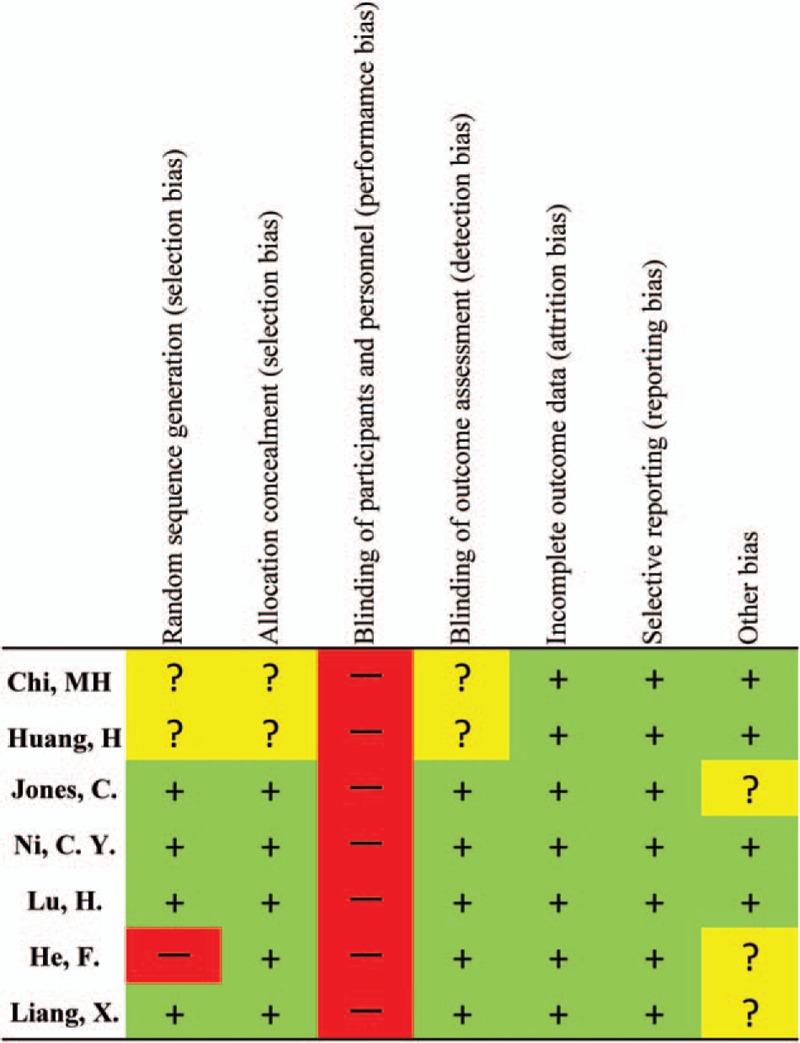
Risk of bias summary in randomized controlled trials (RCTs). The symbol (−) indicates that there is a high risk of bias, (+) indicates a low risk of bias, and (?) indicates uncertainty.

The quality of the 7 RCTs assessed according to the Cochrane Collaboration tool is shown in Fig. 2. Five^[31–35]^ of them were of high quality and 2^[36,37]^ were not clear in random sequence generation, allocation concealment, and blinding of outcome assessment.

### 3.4 Results of meta-analysis

#### 3.4.1 Length of hospital stay

**Figure 3:**
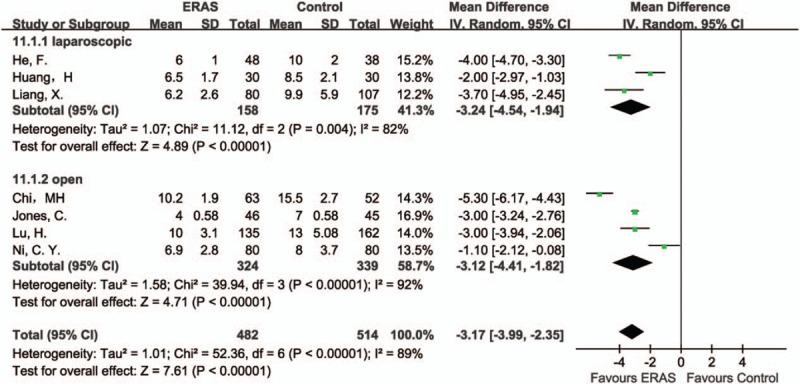
Forest plot of length of hospital stay, comparing ERAS with Control.

All of the 7 studies reported hospital stay. As to laparoscopic liver resection, 3 studies showed a significant result in favor of the ERAS group (MD −3.24, 95% CI: −4.54 to −1.94, *P* < .00001, *I*^2^ = 82%). Similar findings were also found in 4 studies of open surgeries (MD −3.12, 95% CI: −4.41 to −1.82, *P* < .00001, *I*^2^ = 92%). Overall, these results demonstrated a statistically difference between the ERAS and Control groups, in favor of the ERAS group (MD −3.17, 95% CI: −3.99 to −2.35, *P* < .00001, *I*^2^ = 89%) (Fig. 3).

#### 3.4.2 Time to first flatus

**Figure 4:**
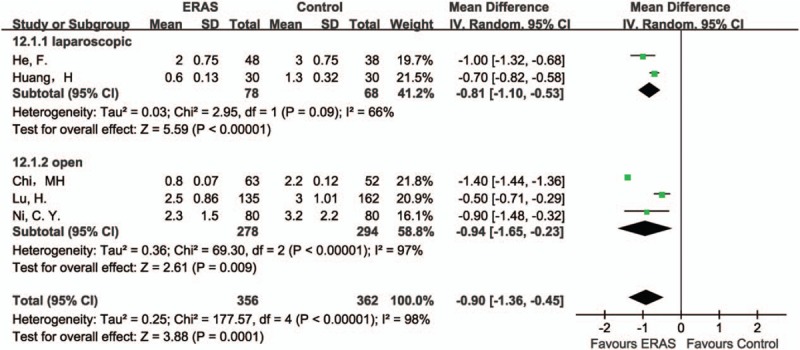
Forest plot of time to first flatus, comparing ERAS with Control.

Five studies^[31,34–37]^ reported time to first flatus. Overall results demonstrated a statistically difference between the ERAS and Control groups, in favor of the ERAS group (MD −0.9, 95% CI: −1.36 to −0.45, *P* = .0001, *I*^2^ = 98%). Similar results were found in both laparoscopic (MD −0.81, 95% CI: −1.10 to −0.53, *P* < .00001, *I*^2^ = 66%) and open surgeries (MD −0.94, 95% CI: −1.65 to −0.23, *P* = .009, *I*^2^ = 97%) (Fig. 4).

#### 3.4.3 Complications

**Figure 5:**
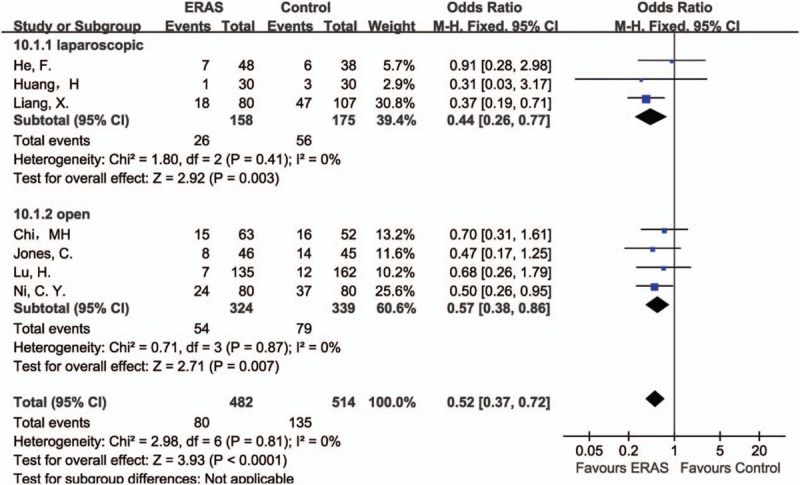
Forest plot of complications, comparing ERAS with Control.

Complications were reported in all the 3 studies of laparoscopic liver resection, and a statistically significant result between the ERAS and Control groups was found, in favor of the ERAS group (OR 0.44, 95% CI: 0.26–0.77, *P* = .003, *I*^2^ = 0%). When compared 4 studies of open liver surgery, the same result was detected (OR 0.57, 95% CI: 0.38–0.86, *P* = .007, *I*^2^ = 0%). Overall, these results demonstrated a statistically difference between the ERAS and Control groups, in favor of the ERAS group (OR 0.52, 95% CI: 0.37–0.72, *P* < .0001, *I*^2^ = 0%) (Fig. 5).

### 3.5 Risk of bias across studies

**Figure 6:**
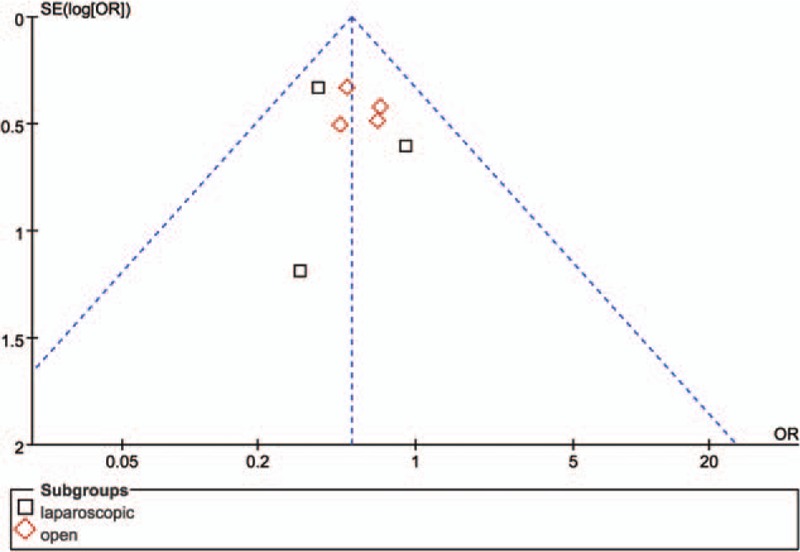
Funnel plot of overall complications, showing no publication bias.

The funnel plot of this study based on overall complications is shown in Fig. 6. All studies lay inside the limits of the 95% CIs and distributed evenly about the vertical, showing no evidence of publication bias.

## 4 Discussion

This review investigated the effects of ERAS on recovery following liver resection. Previous meta-analysis in this area included 5 RCTs representing 723 patients, and concluded that ERAS is a safe and effective program in liver surgery.^[38]^ We optimized search strategies and time limit, and finally included 7 RCTs representing 996 patients in our meta-analysis. We further divided the analysis into laparoscopic and open subgroups to explore the effectiveness of ERAS in different surgical approaches. Therefore, we believe that we can get a more reliable result. Another recent meta-analysis in this field was published in 2016.^[39]^ It included 3 RCTs and 5 cohort studies, representing 810 patients. The majority of their included studies were retrospective, which may lead to more bias. Besides, although it compared more aspects, like restoration of normal diet, functional recovery, and intensive care unit admission rate, we noticed that conclusions from these analyses were only based on 2 or 3 included studies. Given that the raw data in these areas were too small, we did not have a relevant comparison.

In regards to the postoperative outcomes in this meta-analysis, there were obvious reductions of LOS in either pooled analysis or subgroup analysis within the ERAS group. However, discharge criteria varied among included studies. Most of them include: good pain control with oral analgesia only; tolerance of solid food; no intravenous fluids; and mobile independently or at the preoperative level. Besides, some referred to normal or decreasing serum bilirubin,^[32,35]^ but some even did not mention their discharge criteria.^[34,36]^ Therefore, it might affect the power of our analysis.

A significant result in favor of the ERAS group in regards to earlier bowel opening was observed. It might be relevant to avoiding bowel preparation and early oral intake.^[22,30]^ Although there was significant heterogeneity among the trials, similar result was found when a random effect model was applied.

Reduced LOS was usually accompanied by less postoperative complications. Our analysis proved that ERAS was beneficial to decrease postoperative complications after liver resection. As Koea et al^[16]^ reported, decreased perioperative fasting periods, and carbohydrate drinks up to 2 hours before surgery could keep normal blood glucose level and prevent sense of thirst, hunger, and anxiety. In addition, strict intravenous fluids and avoidance of bowel preparation could prevent delayed gastrointestinal function, interstitial edema, lung compliance, and cardiac overload, in order to decrease stress response and complications.^[31,40]^ But because it was hard for surgeons to record every complication accurately and they might be more sensitive to potential complications in the control group, there might be some bias.

As we know, situation of cirrhosis could definitely affect the recovery progress after hepatectomy.^[41,42]^ In the selected studies, only one of them compared preoperative liver function or cirrhosis level.^[33]^ Two studies did not mention American Society of Anesthesiology scores of their included patients,^[36,37]^ and it may also lead to some bias.

Reduced LOS and complications were also accompanied with less hospital costs. He et al^[31]^ showed that the average hospital cost was 9470 ± 1540 in the Control group and only 7742 ± 1200 in the ERAS group (*P* = .03). Page et al^[26]^ reported that introduction of the ERAS pathway was associated with a reduction in hospital-wide costs. For example, a 40.7% decrease in laboratory-associated costs (*P* = .033), a 54.1% reduction in pharmacy-related costs (*P* < .001), and a 21.5% reduction in medical supply costs (*P* = .007). Similarly, reductions in therapy-related costs were found among patients in the ERAS group (*P* < .001).^[26]^ Of note, there were no differences between the 2 groups in operating room or radiology costs.^[26]^

The use of abdominal drains following liver resection was also controversial. Many trials showed it was unnecessary to have routine abdominal drainage,^[43,44]^ while some reports reflected valuable diagnostic and therapeutic benefits of the drains, especially after major hepatectomy.^[45,46]^ Therefore, it might be necessary to have more researches within the ERAS program, comparing strategies with and without routine abdominal drainage.

Many ERAS guidelines have already been established in several kinds of surgeries, such as cystectomy,^[47]^ gastrectomy,^[48]^ and colorectal surgeries.^[49]^ But due to concerns of the specific complications, guideline for liver resection is still not established. Therefore, more high-quality studies in this field are recommended.

Finally, our meta-analysis had several limitations. For example, only 7 RCTs, representing 996 patients were included in our study, the sample size is still limited. Besides, 6 out of the 7 studies were reported in China, which might have geographic bias to a certain degree. And data about pain score, hospital costs, and cirrhosis were insufficient in included literatures. Meanwhile, this meta-analysis only evaluated criteria within one month after surgery. Considering that most of the liver resections were carried out because of malignant tumors, for example, primary hepatocellular carcinoma or metastatic hepatic tumor,^[28,32,33]^ it was important to have long-term oncological outcomes.

## 5 Conclusion

The ERAS program can obviously enhance short-term recovery after liver resection. It is safe and worthwhile. A specific ERAS guideline for liver resection is recommended.
